# Community outbreak of serogroup B invasive meningococcal disease in Beaujolais, France, February to June 2016: from alert to targeted vaccination

**DOI:** 10.2807/1560-7917.ES.2018.23.28.1700590

**Published:** 2018-07-12

**Authors:** Alexandra Thabuis, Karim Tararbit, Muhamed-Kheir Taha, Dominique Dejour-Salamanca, Vincent Ronin, Isabelle Parent du Chatelet, Guillaume Spaccaferri

**Affiliations:** 1 Santé publique France, French national public health agency, Cellule d’intervention en région Auvergne-Rhône-Alpes, Saint-Maurice, France; 2 Agence régionale de santé Auvergne-Rhône-Alpes, Regional health agency, Lyon, France; 3 Institut Pasteur, National Reference Centre for Meningococci, Paris, France; 4 Santé publique France, French national public health agency, Department of Infectious Diseases, Saint-Maurice, France

**Keywords:** invasive meningococcal disease, serogroup B, outbreak, vaccination campaign, 4CMenB/Bexsero

## Abstract

In February and March 2016, four cases of serogroup B invasive meningococcal disease (IMD) occurred over 3 weeks in a small area north of Lyon in the Auvergne-Rhône-Alpes region, France. There were no deaths but two cases had sequelae. This community outbreak was caused by a rare meningococcal strain of the clonal complex ST-32, covered by the 4CMenB/Bexsero vaccine. The incidence rate for serogroup B IMD in this area was 22.5 per 100,000 inhabitants, which is above the epidemic threshold (10/100,000). The number of cases observed was significantly higher than expected in the age group of 0–24 year-olds (standardised incidence ratio: 96). These results suggested the potential emergence of this invasive strain in this sub-population. In accordance with French recommendations, it was decided to vaccinate the population aged between 2 months and 24 years, living, working or studying in the epidemic area. The vaccination campaign took place from April to September 2016. Vaccination coverage was estimated at 47% for one dose and 40% for two doses. The lowest coverage estimations were observed for the age groups younger than 3 and 15–19 years. Enhanced epidemiological and microbiological surveillance reported a fifth case in June 2016, outside the epidemic area.

## Introduction

Invasive meningococcal disease (IMD) is a severe infection caused by *Neisseria meningitidis* which can lead to serious complications and death (the case fatality rate of IMD in France was 11% in 2015) [1]. In France, the IMD notification rate is highest among infants younger than 1 year, followed by children aged 1–4 years and young adults aged 15–24 years. Serogroup B is responsible for more than half of IMD cases, with a case fatality rate of 8% [1].

In France, IMD is a notifiable disease [2]. Following each notification, control measures are implemented by the regional health agency (Agence Régionale de Santé, ARS) to prevent secondary cases. Irrespective of serogroup, chemoprophylaxis is provided to contacts of IMD cases. Unlike serogroups A, C, Y and W, vaccination against serogroup B (with 4CMenB/Bexsero) is not recommended for contacts of sporadic cases. However, it is recommended for target populations in specific situations, such as outbreaks [3]. Outbreaks of serogroup B infections are rare [4-6] and when they occur, the French national public health agency (Santé publique France) may carry out an epidemiological investigation.

In February and March 2016, four cases of serogroup B IMD were reported to the ARS in a small area in Beaujolais, north of the city of Lyon (in the Auvergne-Rhône-Alpes region, France). This number was unusually high and constituted an alert, so an epidemiological investigation was initiated by the local Santé publique France team, in collaboration with the ARS. The aims were to describe characteristics of the cases and their potential epidemiological links, and to suggest control and preventive measures.

## Methods

### Surveillance system for invasive meningococcal disease

IMD cases are notified to the ARS by hospital practitioners through the French IMD surveillance system [1]. The ARS public health team then leads a field investigation (to identify the cases’ sociodemographic characteristics, clinical aspects, activities and contacts during the 10 days before the onset of symptoms) in order to ensure that suitable control measures are applied within the recommended time limits.

Cultured meningococcal isolates and/or primary clinical samples are sent to the National Reference Centre for Meningococci in Paris for full typing. This includes grouping and genotyping using multilocus sequence typing, which defines the sequence type and the clonal complex of the isolates [7]. The typing data are expressed as a combination including group, variable regions VR1 and VR2 of PorA, variable region of the protein FetA and clonal complex. Serogroup B isolates corresponding to clusters are also investigated to measure coverage by the 4CMenB/Bexsero vaccine, either with the meningococcal antigen typing system (MATS) [8] or with serum bactericidal activity using a pool of sera from vaccinated subjects and human complement (hSBA) [9].

### Epidemiological analysis of the Beaujolais outbreak

The epidemiological analysis was conducted by the local Santé publique France team. In line with current French directives [2], the epidemic area was defined as the smallest area covering the place of residence of all four cases. Demographic data were collected from the 2012 national census. Incidence of serogroup B IMD by age group was estimated in this area for the previous 52 weeks and compared with the incidence in the rest of France. The number of expected cases by age group in the epidemic area was estimated using incidence rates of serogroup B IMD in the rest of France (indirect standardisation). The standardised incidence ratio (SIR, i.e. the ratio of observed over expected cases) was calculated for each age group. The epidemic criteria used were those set out by French directives [2]: appearance of at least three cases within 3 months caused by the same strain, occurring without direct contact in the same community, with an incidence rate above 10 per 100,000 inhabitants.

### Decision-making

**Figure 1:**
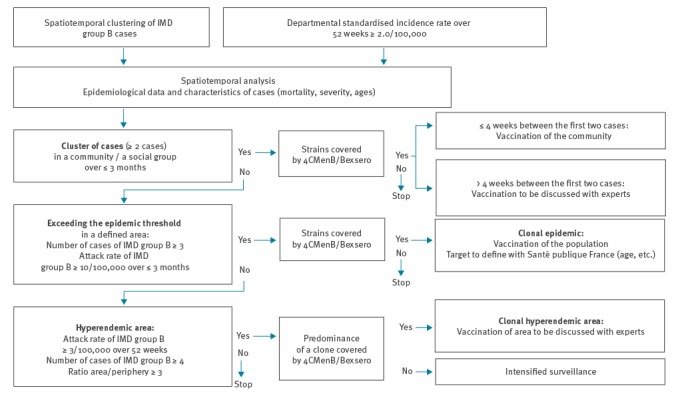
Algorithm for decision-making on vaccination with 4CMenB/Bexsero, serogroup B invasive meningococcal disease outbreak, France, 2016

The decision to vaccinate was taken by the ARS in accordance with the algorithm of decision-making for vaccination with 4CMenB/Bexsero (Figure 1) as set out by French directives [2]. The epidemic area and the target population were defined by an ad hoc multidisciplinary expert committee.

IMD: invasive meningococcal disease.

## Results

### Outbreak description and relationships between cases

Four cases of serogroup B meningococcal meningitis occurred in a small area in France between 29 February and 19 March 2016 (Table 1). A first cluster of two cases (Case 1 and Case 2) involved two 17-year-olds without any direct epidemiological link identified. One month later, a second cluster appeared on the same day in two children aged 3 and 4 years, respectively, who attended the same school class. These two co-primary cases were considered as one event for statistical purposes (Case 3a and Case 3b). While two cases recovered fully, the other two experienced renal sequelae and hearing impairment.

**Table 1:** Description of serogroup B invasive meningococcal disease cases in Beaujolais, France, February–March 2016 (n = 4)

	Cluster 1		Cluster 2 (co-primary cases)	
	Case 1	Case 2	Case 3a	Case 3b
Date of hospitalisation	29 Feb 2016	3 Mar 2016	19 Mar 2016	19 Mar 2016
Age (years)	17	17	4	3
Sex	Male	Female	Female	Female
Symptoms	Meningitis, petechial rash	Meningitis, shock, coma	*Purpura fulminans*, multiple organ failure	Meningitis
Relations between cases	They were present at the same local community ball on 20 Feb 2016, but there was no direct or indirect contact identified between the two cases. No community identified.		They were friends, in the same class at the primary school. Same community identified.	

### Epidemic area

**Figure 2:**
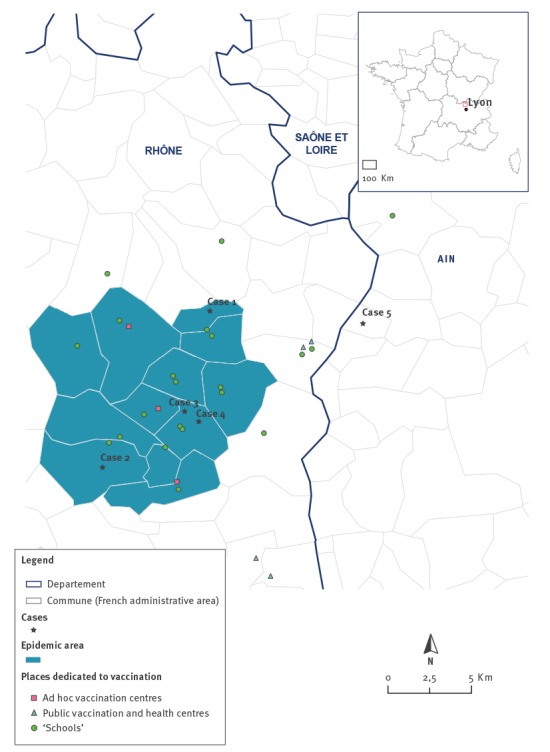
Epidemic area and vaccination centres, serogroup B invasive meningococcal disease outbreak, France, February–March 2016 (n = 4)

Source: Institut national de l'information géographique et forestière (IGN)-GEOFLA, 2016; Santé publique France, 2017.

The smallest area covering the place of residence of all cases was ca 15 km in diameter and comprised 12 communes (French administrative areas) (Figure 2). The epidemic area comprised 13,319 inhabitants, 4,331 of whom were in the age group 0–24 years.

### Incidence rates

For all ages, the incidence rate for serogroup B IMD for the previous 3 months (with co-primary cases counting as one case) was 22.5 per 100,000 inhabitants, which was above the epidemic threshold of 10 per 100,000. The incidence rate for the previous 52 weeks was also 22.5 per 100,000 inhabitants in the epidemic area vs 0.3 per 100,000 in the rest of France (Table 2). For the age group of 0–24 year-olds, the observed number of cases was 96 times higher than the expected number. Given the age of the cases, statistically significant excess cases were observed in the groups aged 0–4 and 15–19 years.

**Table 2:** Cases of serogroup B invasive meningococcal disease and incidence rates by age group for the epidemic area (n = 4) and for the rest of the country (n = 210), France, 21 March 2015–20 March 2016 (52 weeks)

	Incidence rates in the epidemic area			Incidence rates in the rest of France^a^			Estimation of the excess risk in the epidemic area		
Age groups (years)	Number of cases	Population^b^	Incidence per 100,000	Number of cases	Population^b^	Incidence per 100,000	Number of expected cases	SIR	Confidence interval
≤ 4	1^c^	933	107.2	72	3,843,947	1.9	0.017	57	1–319
5–14	0	2,050	0.0	22	7,787,854	0.3	0.006	NA	NA
15–19	2	875	228.6	21	3,818,889	0.5	0.005	416	47–1,501
20–24	0	474	0.00	26	3,828,586	0.7	0.003	NA	NA
≥ 25	0	8,988	0.00	69	44,082,842	0.2	0.014	NA	NA
Total ≤ 24	3	4,331	69.3	141	19,279,277	0.7	0.031	96	19–280
Total	3	13,319	22.5	210	63,362,119	0.3	0.045	66	13–193

NA: not available; SIR: standardised incidence ratio (ratio of observed to expected cases).

^a^ Excluding epidemic area.

^b^ Source: Insee (National Institute of Statistics and Economic Studies), 2012 Census Data.

^c^ The two co-primary cases were counted as one case.

### Laboratory investigations

The cases were confirmed by culture (Case 3b) and PCR (the three other cases). Molecular typing showed that all four cases were caused by *N. meningitidis* isolates with an identical genotypic combination B:P1.19,15:F4–28:cc32 which harboured the penA52 allele.

A MATS analysis was performed on the cultured isolates and predicted that the strain was covered by the 4CMenB/Bexsero vaccine which was expected to be protective based on the level of expression of the gene encoding one of the components of the vaccine, the factor H binding protein fHbp. This prediction was also confirmed by hSBA analysis of the cultured isolate using the pool of sera mentioned in the methods section [9], both before vaccination and 1 month after vaccination with the second dose. This analysis showed a bactericidal titre of 2 in the pool before vaccination and a titre of 128 in the pool after vaccination. This increase in hSBA titre against the outbreak strain was correlated with protection against this strain by the vaccine.

### Control measures around the four cases

The ARS applied control measures according to French directives [2]. Close contacts around the four cases received antibiotic prophylaxis with rifampicin. French directives stipulate that meningococcal B vaccination must also be proposed when a cluster (at least two cases) occurs in an identifiable social group or community over a period of less than 3 months, if the strain is covered by 4CMenB/Bexsero and if the time elapsed between the first two cases is less than 4 weeks (Figure 1). In our outbreak, no identifiable community or social group could be detected for the first cluster (Cases 1 and 2). Therefore no vaccination was performed for the contacts of these cases. For the second cluster (co-primary Cases 3a and 3b), the cases’ school was identified as a community and the ARS immediately organised vaccination for all children and adults (school staff) there.

### Decision-making

The epidemiological analysis of the two clusters showed that all criteria for a clonal epidemic were present. As the strain was covered by the 4CMenB/Bexsero vaccine, this situation corresponded to a clonal epidemic requiring a vaccination campaign after defining the target population (Figure 1). On 1 April 2016, the ARS, together with the multidisciplinary expert committee, decided to propose free vaccination to everyone aged between 2 months and 24 years who were living, were being cared for (by a childminder or in a daycare centre), were studying or working in the epidemic area.

According to the vaccination strategy using 4CMenB/Bexsero (two to four injections according to age), the number of doses necessary to vaccinate the target population was estimated at ca 9,000. The ARS covered the costs of the campaign. A contract with the local health insurance fund reimbursed the ARS 65% of the costs of the vaccines. The expert committee also decided to enhance epidemiological and microbiological surveillance in this region of France for at least 1 year.

### Vaccination campaign

Vaccination against the *N. meningitidis* serogroup B is not part of the current French immunisation schedule. Accordingly, the quantity of 4CMenB/Bexsero vaccine administered is low in France (except during epidemics) and pharmacies generally have no stock. Therefore, the vaccine was not readily available in sufficient amounts at the beginning of the campaign. To overcome this, the ARS first procured the doses available from pharmaceutical wholesaler-distributors to offer them to the vaccination centres, and then ordered the remaining quantity of vaccine doses directly from the pharmaceutical company. During the initial period (from 5 April to 24 June 2016), the ARS organised vaccination sessions in various locations (Figure 1): ‘schools’ (daycare centres, primary and secondary schools) in and around the epidemic area, ‘ad hoc vaccination centres’ in the epidemic area (premises provided by local councils) and ‘public vaccination and health centres’ in and around the epidemic area. During the subsequent period (from 1 June to 30 September 2016), the ARS managed to make 4CMenB/Bexsero more widely available in 12 pharmacies serving the epidemic area, so that general practitioners were able to vaccinate people in their private practices.

General information on the campaign was diffused through official ARS press releases and newsflashes broadcast by local radio stations. The campaign also received coverage by local newspapers. The target population received printed information letters directly in their mailboxes, in schools, in daycare centres and from childminders. Posters and flyers were displayed in pharmacies, private practices, public vaccination centres/health centres and public places. A toll-free hotline was also set up to provide information and the ARS website was regularly updated. Parents of school-age children were invited to an information meeting before each vaccination session organised in schools. Local health workers were invited to specific information meetings throughout the campaign.

### Vaccination coverage estimates

During the campaign, the local Santé publique France team monitored the number of vaccinations in real time, in order to estimate vaccination coverage and adapt the offer of vaccination. At the end of the campaign, 4,062 vaccinations had been administered (2,222 first doses and 1,840 second doses). Considering only persons who lived inside the epidemic area, mean vaccination coverage estimates were 47% for one dose (n = 2,038 doses) and 40% for two doses (n = 1,716) (Table 3). The lowest estimates were for those younger than 3 years and those aged 15–19 years.

**Table 3:** Vaccination coverage estimates among those ≤ 24 years of age in the epidemic area, serogroup B invasive meningococcal disease outbreak, France, 2016 (n = 4,338)

Age groups (years)	Estimated target population	Vaccination coverage estimates	
		1 dose	2 doses
< 3	544	38%	30%
3–11	1,847	70%	63%
12–15	828	44%	36%
16–24	1,119	14%	8%
Total ≤ 24	4,338	47%	40%

### Pharmacovigilance follow-up

A reinforced pharmacovigilance follow-up was set up in collaboration with the regional pharmacovigilance centre in Lyon. A specific questionnaire filled in by parents and general practitioners was used for data collection. Of 4,062 first and second doses administered, 152 notifications were received (3.7 notifications per 100 administered doses) and 81% reported at least one local or loco-regional reaction. In total, 309 adverse effects were described. Among these, none was considered severe according to the World Health Organization (WHO) criteria of severity; four were considered medically relevant, one of which was deemed unexpected (i.e. generalised rash) in terms of current knowledge about the 4CMenB/Bexsero vaccine.

### Enhanced surveillance

During the year of enhanced surveillance, one additional case caused by the same *N. meningitidis* strain was notified in the same region. This fifth case was reported on 28 June 2016. It occurred in a 14-year-old boy who lived ca 10 kilometres outside the epidemic area, without any direct link to the outbreak area. This case occurred more than 3 months after the third and fourth cases, therefore the new incidence rate for the previous 3 months did not exceed the epidemic threshold. The alert was officially lifted on 1 July 2016 at a meeting of the expert committee. After this fifth case, no other case caused by the same strain was reported in this region.

## Discussion

Between February and March 2016, a clonal epidemic of IMD caused by a very rare strain of *N. meningitidis* serogroup B occurred in a small area located in Beaujolais, north of Lyon. Excess cases were observed in the age groups 0–4 and 15–19 years. This situation indicated the possible emergence of this strain in a non-immune population. To prevent further cases and to stop the strain from gaining a foothold in this area, an expert committee decided to implement vaccination. In line with current French directives, 4CMenB/Bexsero vaccine was proposed to those aged from 2 months to 24 years who were living, studying and working in the epidemic area. Vaccination coverage was estimated at 47% for the first dose and 40% for the second.

All cases were caused by *N. meningitidis* isolates with an identical genotypic combination B:P1.19,15:F4–28:cc32 which harboured the penA52 allele. Isolates belonging to the hyperinvasive clonal complex cc32 are frequent in France and represented 26% of all invasive serogroup B cases during the period from 2006 to 2015 [10]. Isolates of cc32 were also responsible for the hyperendemic situation in Normandy (2003–2012) which was controlled by vaccination using MenBvac [4]. However, the combination of markers B:P1.19,15:F4–28:cc32 with the additional marker penA52 is rare among the cc32 isolates in France, with only three other cases being reported since 2009 in different regions of the country. This would suggest that this genotypic combination has a low circulation. The cluster reported in this study may therefore indicate a recent introduction of this strain in a naive population in Beaujolais. Similar isolates were reported in the United Kingdom (UK) during between 2010 and 2012, but they were rare, just like in France [11]. Moreover, the finetype B:P1.19,15:F4–28:cc32 is rare in Europe with a total of only 26 isolates being reported between 2009 and 2016 in France, Lithuania, Spain and the UK on the PubMLST.org website.

The vaccination campaign with 4CMenB/Bexsero described here was the first in France since the drug was licensed (January 2013). In France, 4CMenB/Bexsero is not included in the national immunisation schedule. Its utilisation must comply with government recommendations [12] and the quantity of available doses is limited.

As there are no data currently available to assess the impact of 4CMenB/Bexsero on meningococcal carriage [13], the vaccination had no coverage objective set. The primary goal was to guarantee individual protection. The highest vaccination coverages were achieved for school children aged 3–11 and 12–15 years. This may be explained by the fact that vaccination sessions were organised directly inside schools (no specific involvement of the families was required) and immediately after the public health alert.

The lowest vaccination coverage was reached in the two age groups most impacted by IMD (0–3 and 16–24 years). The result in 16–24-year-olds was not surprising given that young adults are difficult to reach and to convince about vaccination. Similar results with a lower coverage in young adults have been observed with the vaccine against serogroup C IMD in France [14-17].

The result for children under 3 years is more unexpected. The vaccination coverage against serogroup C IMD was 67% for the 2–4-year-olds in this region in 2013 [14]. This may reflect a lack of acceptance by some healthcare professionals who are unfamiliar with the vaccine or who feel that there is limited information on the duration of protection [13].

In order to analyse the precise reasons for vaccination or non-vaccination in this campaign, a psycho-sociological study was commissioned by the ARS which showed that the target population had been well informed about the campaign. Indeed, 87% of respondents had read the information letter and 94% had discussed it with friends or family. Parents who did not vaccinate their children felt less concerned by the risk of IMD and were less sure about the efficacy or safety of the vaccine. As a result, psycho-sociologists suggested that in future campaigns for IMD epidemics, the ARS should insist more on the substantial threat of transmission and provide greater reassurance about the vaccine’s efficacy and safety (personal communication, Maéva Bigot, May 2017).

Tolerance of 4CMenB/Bexsero was good during this campaign. The adverse effects reported were conform to the summary of the product’s characteristics and no severe adverse effects (according to the WHO definition) were observed. This information is reassuring as 4CMenB/Bexsero was used for the first time in such a campaign in France. Although the number of notifications of adverse effects (n = 152) may appear low with respect to the number of doses administered (3.7 notifications per 100 administered doses), one can hypothesise that under-reporting rather concerned minor effects while more serious effects were reported more exhaustively.

The fifth case occurred more than 3 months after the last outbreak case. Vaccination was not proposed to this case because he lived in a commune located outside the defined epidemic area (i.e. the smallest homogeneous area including all cases and taking into account the concept of ‘catchment area’). If a larger area including this commune had been defined, the area would have comprised more than 40,000 inhabitants and the incidence rate would have been less than the epidemic threshold of 10 per 100,000 inhabitants.

## Conclusion

The action plan employed to manage this outbreak was in line with current French directives: alert, type strains, test vaccine coverage, decide to vaccinate and define the target population, implement the vaccination campaign, follow up vaccination coverage and pharmacovigilance, reinforce epidemiological and microbiological surveillance. This method, which helped prevent the spread of this strain, constitutes a reproducible approach.
